# Bilateral sinonasal inverted papillomas originating from both sides of the frontal sinus and the left lamina papyracea: A case report

**DOI:** 10.1097/MD.0000000000037703

**Published:** 2024-04-12

**Authors:** Yang Li, Dengdian Ma

**Affiliations:** a School of Clinical Medicine, Jining Medical University, Shandong, Jining, China; b Department of ENT, Affiliated Hospital of Jining Medical University, Shandong, Jining, China.

**Keywords:** bilateral, case report, ethmoid sinus, frontal sinus, sinonasal inverted papilloma

## Abstract

**Rationale:**

The present investigation documented a case of bilateral sinonasal inverted papilloma (SNIP) that arose from both sides of the frontal sinus and ethmoid sinus. The occurrence of bilateral involvement of the nasal cavities and frontal sinus is rather infrequent.

**Patient concerns:**

Informed consent was obtained from the patient.

**Diagnosis:**

Bilateral SNIP.

**Interventions:**

The tumor was completely removed by Draf III endoscopic resection complemented by an external eyebrow arch approach, and the postoperative recovery was uneventful.

**Outcomes:**

The purpose of this paper is to present a comprehensive reference for the management of bilateral SNIP that affects the frontal sinuses.

**Lessons:**

This study addresses the staging and surgical management of bilateral SNIP, along with a review of the factors contributing to its recurrence. The recommended treatment method involves applying the Draf III technique combined with an external nasal approach.

## 1. Introduction

Sinonasal inverted papilloma (SNIP) is a commonly occurring nasal tumor originating from well-differentiated epithelial cells within the smooth connective tissue.^[1]^ SNIPs exhibit distinctive clinicopathological characteristics, such as a high recurrence likelihood, a propensity for local invasiveness, multicentricity, and the potential for developing carcinogenic changes. The majority of patients diagnosed with SNIPs exhibit unilateral tumors, whereas cases with bilateral SNIPs are rather infrequent, accounting for a range of 0% to 5% of all reported instances.^[2]^ Moreover, SNIPs are typically located in the lateral wall, followed subsequently by the maxillary and ethmoid sinuses.^[3]^ Consequently, the occurrence of bilateral SNIPs in the frontal sinus is regarded as extremely uncommon. In this study, a patient with recurrent bilateral SNIPs that originated from both sides of the frontal sinus and the left lamina papyracea was reported. The patient underwent a therapeutic intervention known as the Draf III treatment, which was performed in conjunction with an external approach. The prognosis for this patient was uneventful.

## 2. Case Presentation

**Figure 1.**
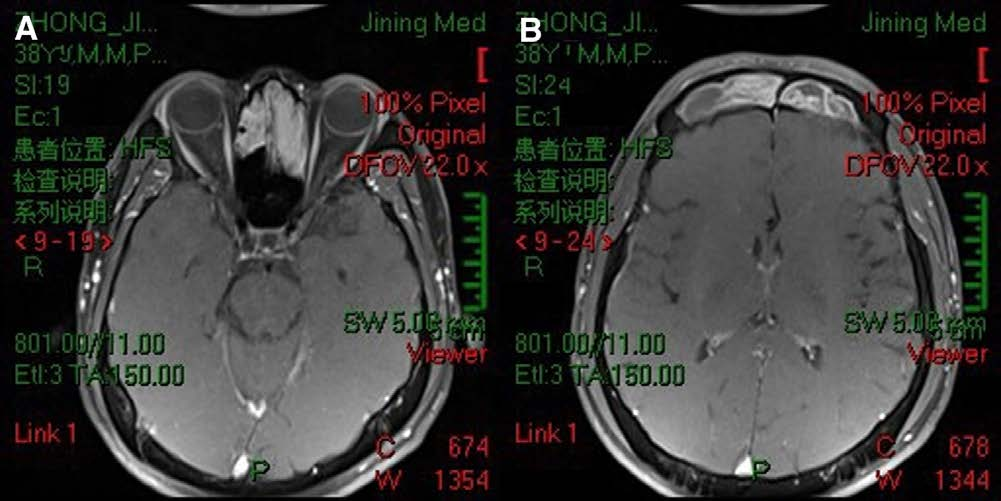
Preoperative MRI scan. (A) Bilateral ethmoidal sinuses and (B) bilateral frontal sinuses occupied by sinonasal inverted papilloma. MRI = magnetic resonance imaging.

**Figure 2.**
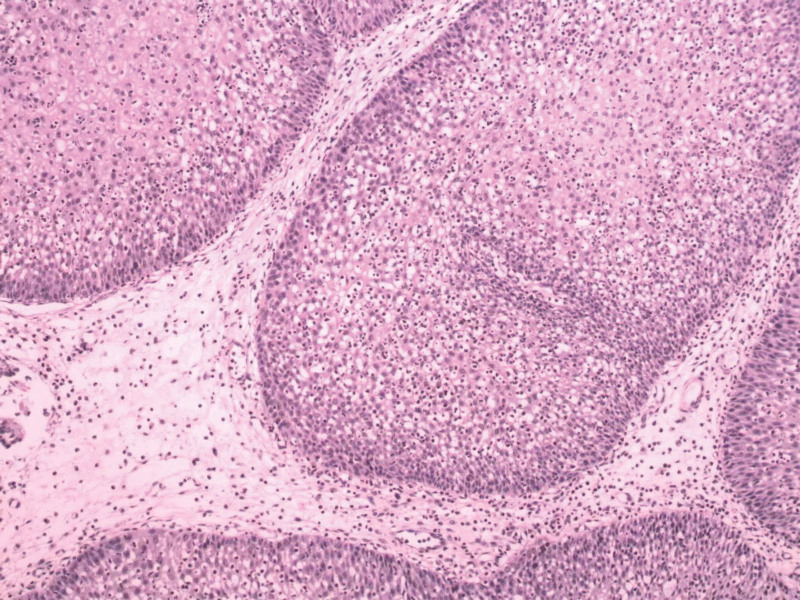
Grayish white fragmented tissue, with a size of 5*4*2 cm; the soft texture was seen by the naked eye. The pathological diagnosis was bilateral nasal inverted papilloma (prone to recurrence).

**Figure 3.**
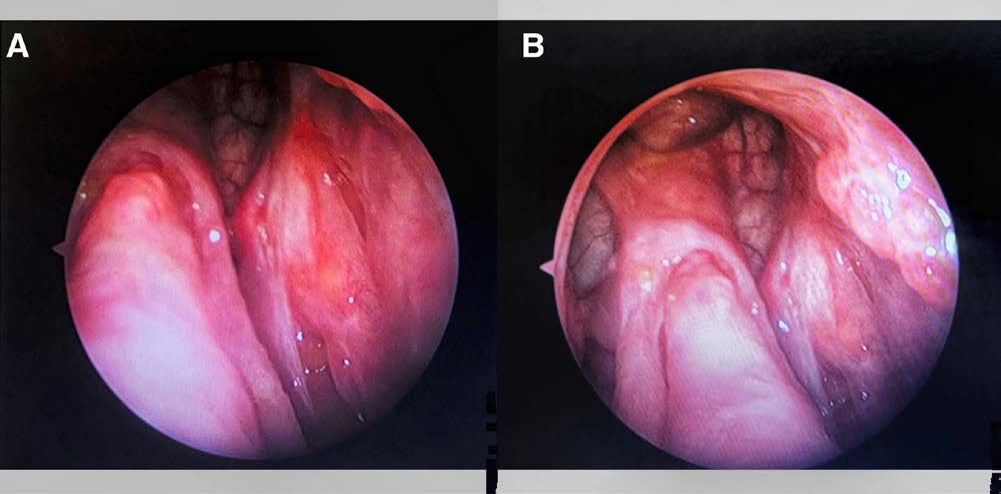
Postoperative nasal endoscopic examination. The bilateral nasal cavities showed post-nasal endoscopic changes with good opening of all sinus.

The patient, a male individual aged 38, presented with persistent symptoms of rhinorrhea and nasal obstruction, along with hyposmia and occasional headaches, which have been ongoing for a duration of 3 years. Following an otorhinolaryngological assessment, the patient was subsequently hospitalized due to the presence of a nasal sinus tumor and chronic rhinosinusitis, leading to a recommendation for surgical intervention. The individual underwent a surgical procedure known as nasal inverted papilloma (IP) excision approximately 4 years ago. No previous record of genetic diseases or any familial history of associated illnesses was documented. The results of the physical examination indicate a generally favorable state of health. Magnetic resonance imaging (MRI) was performed, revealing the presence of a tumor in the nasal sinus (as depicted in Fig. 1A and B). The histological examination indicated the presence of bilateral SNIP (Fig. 2). The patient diagnosis was determined to be a recurring nasal sinus mass and chronic rhinosinusitis, based on the results of the experiment. According to the therapeutic criteria, the recommendation for the Draf III procedure was made due to the identification of substantial invasion in both the ethmoid sinus and frontal sinus, which was classified as Krouse stage III. The nasal endoscopic Draf III frontal sinus opening, combined with an extra-nasal method known as the arch incision approach, was conducted on June 1,2015, while the patient was under intravenous anesthetic. A rapid intraoperative pathological diagnosis confirmed the presence of an IP in the sinus. The nasal endoscopic procedure provided evidence that the tumor stemmed from the left lamina papyracea and the frontal saphenous fossa on the opposite side. The tumor was surgically separated along the left lamina papyracea. Moreover, it was extracted from the frontal fossa by a grinding technique applied to the frontal maxillary process on both sides, directed toward the frontal nasal ridge. This procedure also involved the removal of a portion of the middle and upper nasal septum, extending into the bilateral frontal sinus orifices. Furthermore, surgical intervention involved the resection of both sides of the frontal sinus floor, as well as a segment of the frontal maxillary process. A surgical procedure involved creating an incision in both brow arches and the anterior wall of the frontal sinus, with the objective of partially excising the tumor located within the frontal sinus. The neoplasm was observed to have its source in the septum of the frontal sinus, as well as the inferior region of the frontal sinus in conjunction with the left supraorbital wall. Following surgery, the nasal cavities were filled with budesonide solutions and absorbable gelatin sponges, therefore concluding the operation. A tissue sample obtained from the tumor root was subjected to regular pathological examinations through an intraoperative frozen section procedure. The findings validated the presence of bilateral SNIPs. After the surgical procedure, the patient vital signs remained steady. In accordance with the Chinese guidelines for the diagnosis and treatment of chronic rhinosinusitis, the patient adhered to a treatment regimen consisting of nasal glucocorticosteroids and oral mucus promoters for a minimum duration of 3 months following surgery. Additionally, nasal saline rinses were used as a supplementary measure. The patient experienced an uneventful surgical recovery, and follow-up nasal endoscopy was initiated at week 1, with subsequent evaluations scheduled at 5-month intervals. Postoperative alterations and the presence of well-opened sinuses were detected in both nasal cavities, with no evidence of recurrence (Fig. 3A and B).

## 3. Discussion

SNIPs commonly manifest in the lateral wall of the nasal cavity and can subsequently extend to the maxillary, ethmoid, or sphenoidal sinuses, as well as the frontal sinus to a lesser degree.^[2]^ The likelihood of such a spread is approximately 6.5%.^[4]^ Furthermore, it is worth noting that the occurrence of primary bilateral sinuses involvement is exceptionally rare, with a prevalence ranging from 1.9% to 2.4%.^[5,6]^ Hence, the occurrence of bilateral IPs originating from the ethmoid and frontal sinuses is regarded as uncommon.

The patient was admitted to the hospital because of SNIPs and underwent a comprehensive preoperative MRI and examination of pathology slides. The surgical treatment chosen for this case was nasal endoscopic surgery in conjunction with an arch incision. Following the establishment of the surgical approach, the tumor was meticulously and thoroughly dissected. The patient exhibited a low level of bleeding, and the prognosis was favorable.

Krouse devised a staging approach that incorporates endoscopic and imaging results, providing a comprehensive assessment of surgical effectiveness by evaluating tumor invasion and potential malignancy.^[7]^ This staging system has gained significant traction in clinical settings and is extensively employed. The MRI scan findings indicated bilateral involvement of the ethmoid and frontal sinuses, which is consistent with a Krouse stage III SNIP. The functional endoscopic sinus surgery-assisted Draf III procedure is commonly recommended for patients with T3 IPs.^[8]^ In cases where the tumor is unable to be surgically eliminated due to anatomical limitations, the utilization of endoscopic surgery would not be considered a suitable treatment approach. Therefore, the methodology might be transformed into a combination of endoscopic and external approaches. Nevertheless, several studies have documented the limitations of this system in accurately forecasting the likelihood of reoccurrence.^[9]^ Through an analysis of the tumor root site and the recurrence rate in patients with SNIP, the Beijing Tongren team has established a novel staging system and provided advice on surgical method selection.^[10]^ Patient in this study can be placed in stage 3 according to Beijing Tongren staging system.^[11]^ As a result, the ESS-assisted Draf III treatment was advised, which was consistent with our surgical strategy. The integration of the Krouse staging system with the Beijing Tongren staging system allows for an in-depth assessment of SNIP lesions, resulting in a more precise surgical approach and improved prognosis for patients.

Based on prior research, the endoscopic endonasal method offers noteworthy advantages such as the absence of external lesions, reduced surgical duration, decreased intraoperative bleeding, fewer complications related to anesthesia, diminished postoperative pain, and a lower occurrence of mucocele formation.^[4,12]^

The performance of endoscopic surgery for SNIP involving frontal sinuses presents major challenges, necessitating a tailored surgical technique that considers the tumor attachment, extension, and the unique anatomical characteristics of each sinus. Professor Draf, hailing from Germany, has categorized endoscopic frontal sinus surgery into 3 distinct categories, namely Draf I, Draf IIa/Draf IIb, and Draf III.^[3,8]^ Regarding the aforementioned 2 staging systems, it is observed that when the root of SNIP is situated in the medial region of the midline of the bilateral pupils, the tumors can be effectively excised through the use of Draf IIb or Draf III endoscopic nasal surgery techniques. In cases when severe frontal sinus lesions are present and the extent of the tumor extends beyond the lateral aspect of the vertical midline of the pupil, the Draf III technique combined with a brow arch incision is advised.

Among the various nasal endoscopic techniques available, the Draf III treatment is the optimal surgical choice for this situation. Additionally, this surgical approach is especially beneficial in cases where there are bilateral tumors localized in the frontal sinus. The main task of the Draf III treatment is to excise the underlying walls of the frontal sinuses on both sides, as well as the surrounding nasal septum, to establish an unobstructed frontal sinus drainage pathway.

Nevertheless, accessing certain places via endoscopy might pose challenges, such as the lateral wall of the frontal sinus, owing to the intricate anatomical structures and the limited vision field provided by the 70°-angled endoscope.^[13]^ The full removal of tumors becomes especially difficult when dealing with well-pneumatized frontal sinuses. A combined bilateral brow arch incision is recommended in this circumstance in order to thoroughly expose and eliminate the tumor.^[14]^ The Draf III procedure combined with an external nasal approach seems a favorable treatment option for IPs involving the frontal sinus attachment, with a low recurrence rate and less morbidity of postoperative complications.^[14,15]^ Furthermore, the successful execution of the Draf III frontal sinus surgery demands the utilization of high-quality technical equipment and the provision of sufficient training. It is noteworthy to emphasize that the combined surgical procedure is characterized by invasiveness, resulting in a substantial postoperative lesion, the potential occurrence of forehead numbness, and the requirement for extended periods of follow-up. Research has been conducted to prevent numbness by bypassing the nerve during surgery based on the vascular alignment of the arch of the eyebrow.

According to the Krouse classification system, it has been discovered that IPs classified at stage T3 exhibit a 51% increased likelihood of relapse. When surgery is suggested for T3 SNIPs, surgeons should pay more attention to this elevated risk of recurrence.^[16]^ In addition, it should be acknowledged that the rate of recurrence was considerably greater in individuals who underwent revision procedures compared to those with original lesions. The existence of a previous surgical history can be regarded as a potential risk factor. At least 5 years of long-term follow-up are necessary for these patients.^[6,17]^ The observed recurrence rate in the combined approach group was found to be 12.9%.^[18]^ To mitigate the risk of tumor recurrence, it is essential to excise the tumor meticulously and thoroughly during the initial surgical procedure, along with appropriate postoperative monitoring. Whether or not the tumor is completely resected, a previous history of surgery can be a decisive factor in recurrence. Furthermore, it is of utmost need to locate the bone to which the IP attaches during the operation, given that the IP is mostly underlain by abnormal bone and its abundant peripheral vascular supply.^[19]^

In addition to its high recurrence rate, IP may also induce a high risk of carcinogenic alterations after a prolonged period.^[20]^ Following the surgical removal of a tumor, it is possible to detect other lesions, such as latent cancer cells or micro-metastases, in a relatively short period of time.^[21]^ Hence, patients who have undergone revision surgery display a higher incidence of metachronous malignancy.^[22]^ Finally, given the potential for recurrence and carcinogenesis, it is advisable to perform long-term follow-up for IP patients.

## 4. Conclusion

This report is a case study of a patient with bilateral SNIP originating from both sides of the frontal sinus and the left lamina papyracea. The endoscopic procedure combined with an external eyebrow arch approach was performed properly based on the previous imaging study of tumor boundaries. Based on that, the optimal approach can be employed, resulting in to the surgical resection of tumors and a reduction in the likelihood of tumor recurrence.

**Table:** TIMELINE

Date	Summaries from initial and follow-up visits	Diagnostic testing	Intervention
2011	Sinonasal Inverted Papilloma	Unknown	The nasal inverted papilloma excision
June 1, 2015	Bilateral sinonasal inverted papillomas originating from both sides of the frontal sinus	MRI and histological examination showing bilateral SNIP	The Draf III technique combined with an external nasal approach
2017	No evidence of recurrence.	Nasal endoscopy	None

MRI = magnetic resonance imaging, SNIP = sinonasal inverted papilloma.

## Acknowledgments

We would like to acknowledge the reviewers for their helpful comments on this paper.

## Author contribution

**Investigation:** Yang Li.

**Writing – original draft:** Yang Li.

**Writing – review & editing:** Yang Li, Dengdian Ma.
